# Genomic Islands of Speciation in *Anopheles gambiae*

**DOI:** 10.1371/journal.pbio.0030285

**Published:** 2005-08-09

**Authors:** Thomas L Turner, Matthew W Hahn, Sergey V Nuzhdin

**Affiliations:** **1**Center for Population Biology, University of California, Davis, California, United States of America; University of EdinburghUnited Kingdom

## Abstract

The African malaria mosquito, *Anopheles gambiae* sensu stricto *(A. gambiae),* provides a unique opportunity to study the evolution of reproductive isolation because it is divided into two sympatric, partially isolated subtaxa known as *M* form and *S* form. With the annotated genome of this species now available, high-throughput techniques can be applied to locate and characterize the genomic regions contributing to reproductive isolation. In order to quantify patterns of differentiation within *A. gambiae*, we hybridized population samples of genomic DNA from each form to Affymetrix GeneChip microarrays. We found that three regions, together encompassing less than 2.8 Mb, are the only locations where the *M* and *S* forms are significantly differentiated. Two of these regions are adjacent to centromeres, on Chromosomes 2L and X, and contain 50 and 12 predicted genes, respectively. Sequenced loci in these regions contain fixed differences between forms and no shared polymorphisms, while no fixed differences were found at nearby control loci. The third region, on Chromosome 2R, contains only five predicted genes; fixed differences in this region were also verified by direct sequencing. These “speciation islands” remain differentiated despite considerable gene flow, and are therefore expected to contain the genes responsible for reproductive isolation. Much effort has recently been applied to locating the genes and genetic changes responsible for reproductive isolation between species. Though much can be inferred about speciation by studying taxa that have diverged for millions of years, studying differentiation between taxa that are in the early stages of isolation will lead to a clearer view of the number and size of regions involved in the genetics of speciation. Despite appreciable levels of gene flow between the *M* and *S* forms of *A. gambiae*, we were able to isolate three small regions of differentiation where genes responsible for ecological and behavioral isolation are likely to be located. We expect reproductive isolation to be due to changes at a small number of loci, as these regions together contain only 67 predicted genes. Concentrating future mapping experiments on these regions should reveal the genes responsible for reproductive isolation between forms.

## Abstract 2

Using DNA microarrays, the authors identify 3 small regions of the genome that differ between two forms of hybridizing mosquitoes; regions that are likely to contain the genes responsible for reproductive isolation.

## Introduction

Uncovering the genetic basis for reproductive isolation is a key to understanding how biological diversity is generated. Many researchers have used quantitative trait locus (QTL) mapping experiments to find the number and size of regions involved in both pre- and post-mating isolation between species (e.g., [1–6]). Although QTL mapping experiments are a powerful method for mapping large regions of the genome responsible for isolation traits, large numbers of recombinant offspring or advanced genetic tools are needed to fine-map the genes underlying QTLs (e.g., [7–9]). By contrast, studies of genomic differentiation between naturally hybridizing taxa make it possible to take advantage of the many recombination events that occur between backcrossed hybrid individuals in order to map the regions responsible for reproductive isolation [10–14].

*Anopheles gambiae* sensu stricto *(A. gambiae),* is the type species of the *Anopheles gambiae* sensu lato complex: a group of seven closely related African species morphologically indistinguishable as adults [15] and incompletely reproductively isolated from one another (hybrid females are fertile) [15,16]. The observation that some species blood-feed exclusively on humans and breed in artificial environments (which have been available for less than 10,000 y) further suggests that this species complex is the result of recent radiation [17]. In addition to these seven recognized taxa, *A. gambiae* is further subdivided into two partially isolated taxa known as the *M* form and *S* form [18]. These forms were originally delineated based on several tightly linked single nucleotide polymorphisms (SNPs) in the rDNA of the X chromosome that are rarely found as heterozygotes [19]. Subsequent studies of reproductive isolation in nature revealed that these forms mate assortatively, with 98.8% of wild-caught gravid females (within an area of sympatry) having mated within their own form [20,21]. When forms are crossed in the lab, no intrinsic fitness reductions are found [22], suggesting that the observed heterozygotic deficiencies are due to nonrandom mating and/or ecologically dependent postzygotic isolation. Studies of gene flow using microsatellite markers have repeatedly found no appreciable genetic differentiation outside the centromeric end of the X chromosome (except between inversions that are not fixed between forms [23]), but these studies have only genotyped 10–25 loci [23–27].

To better examine the genetic basis for the maintenance of reproductive isolation between the *M* and *S* forms of *A. gambiae* and to delineate the number and size of regions that do not introgress—which may contain genes involved in reproductive isolation—we hybridized DNA of single mosquitoes from population samples of *M* and *S* forms to Affymetrix GeneChip microarrays. Recent studies in *Saccharomyces cerevisiae* [28] and *Arabidopsis thaliana* [29] have shown that hybridizing genomic DNA to Affymetrix arrays, which are printed with 25 base pair (bp) oligonucleotides (“probes”), allows precise mapping of DNA polymorphisms between samples. Because the hybridization intensity between probes on the array and DNA in the sample depends on sequence identity, polymorphisms located within these probes (either single nucleotide differences or small indels) can be quantified. Borevitz et al. [29] used this technique to genotype two *Arabidopsis thaliana* inbred strains and their recombinant inbred line, and were able to precisely delineate which regions of the recombinant line came from either parental line. Our goals were (1) to locate regions of the genome where *M* and *S* forms differed; (2) to test whether the observed pattern of differentiation resulted from selection against gene flow or could be explained by genetic drift or other processes; and (3) to determine what this genomic pattern could tell us about speciation and adaptive radiation.

## Results

We used seven samples of *M* form and seven samples of *S* form mosquitoes from areas of Cameroon where they are sympatric (see Materials and Methods), and where gene flow at microsatellites is known to be high [26]. These samples were chosen to avoid the confounding factor of several segregating inversions found within *A. gambiae* [23,30]. In Cameroon both *M* and *S* forms possess the same (standard) karyotype [26].

We remapped each 25-bp probe on the Affymetrix *Plasmodium/Anopheles* GeneChip array to the most recent *A. gambiae* genome assembly and removed probes with multiple exact matches, which generated a marker map of 142,065 unique probes (see Materials and Methods). Whole genomic DNA from single female mosquitoes was hybridized to each array, with seven individuals hybridized per form (a total of 14 arrays). In addition, one mosquito DNA isolate was labeled and hybridized a second time as a technical replicate. As expected, all samples were very similar, with the technical replicates more highly correlated than any of the biological replicates, indicating high reproducibility (average Spearman *M* vs. *M* correlation = 0.954, average Spearman *M* vs. *S* correlation = 0.942, Spearman technical replicate correlation = 0.989).

**Figure 1:**
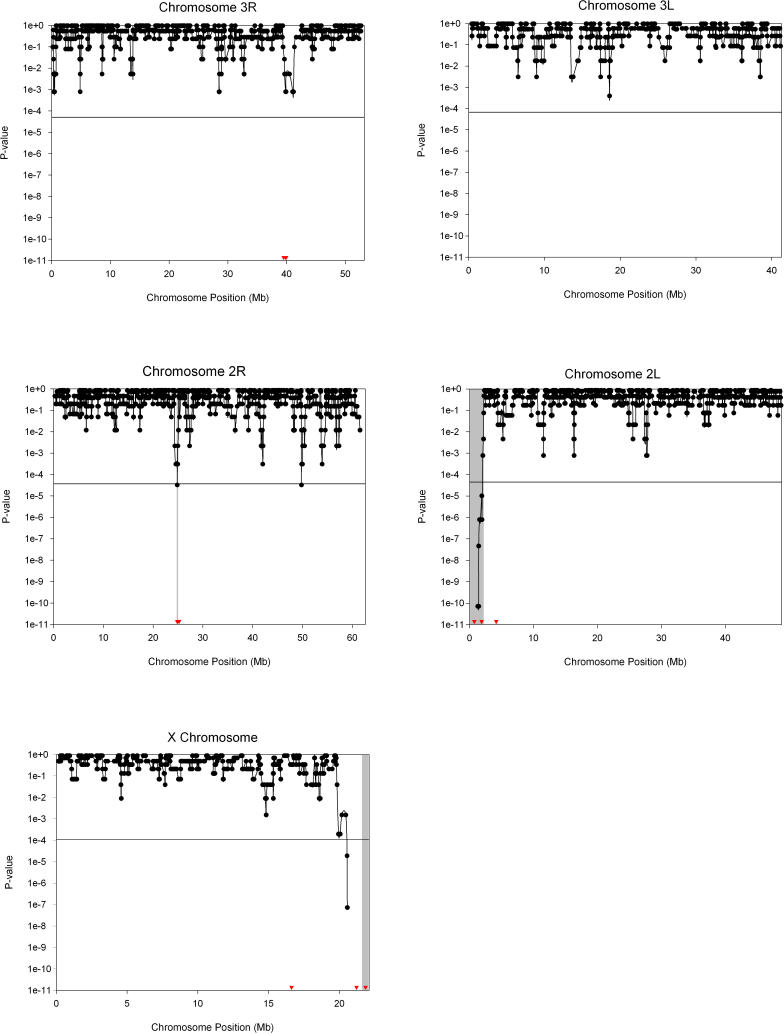
Differentiation between Forms The significance threshold shown is *p* = 0.05, Bonferroni-corrected for the number of windows tested per chromosome. The centromeres of Chromosomes 2 and 3 are located between the right and left arms, i.e., between each pair of graphs; the centromere of Chromosome X is located at the right end of the graph. Grey areas are divergent regions identified by our HMM. The grey region at the tip of Chromosome X appears to lie outside of the final window because the chromosomal position given for each window is the location of the central probe in that window; the final window on Chromosome X spans a large region because of low gene density. Sequenced loci are shown with red triangles; overlapping triangles on Chromosomes 3R and 2R obscure multiple sequenced loci (see text for details). 3L, left arm of Chromosome 3; 3R, right arm of Chromosome 3.

Differentiation between forms is shown in Figure 1. To predict which probes contained polymorphisms between forms, we calculated *t-*test *p-*values for each probe and considered probes with *p* < 0.01 to be candidate single-feature polymorphisms (SFPs; [29]). We also directly sequenced several candidate SFPs to verify these differences (see below and Materials and Methods). The number of probes with differences between forms within a window of 300 probes was then tested against the number expected to appear if sequence differences were distributed randomly across each chromosome (via a χ^2^ test). The null hypothesis in this analysis, a random distribution of SFPs, could be violated simply because of linkage disequilibrium of probes within a gene. We permuted probesets—preserving the association of probes within a gene—to test for this effect (see Materials and Methods). After correcting for multiple tests, four regions were found to be significantly differentiated in the initial sliding window analysis: the region proximal to the centromere on the left arm of Chromosome 2 (2L), the centromeric end of the telocentric X chromosome, and two regions on the right arm of Chromosome 2 (2R). We also searched for differentiated regions using a hidden Markov model (HMM), which recovered the regions on Chromosomes 2L and X, and one of the regions on Chromosome 2R (see Materials and Methods). The 2L and X regions remained highly significant after permutation testing (2L, *p* = 0.002; X, *p* < 0.001), but the 2R regions were not significant. For the 2R region detected in both analyses, nonsignificance may be due simply to its small size in relation to the size of sliding windows: seven of 11 SFPs in this window fell within four probesets, spanning only 40 kilobases (kb). Overall, the significance of the differentiated regions on Chromosomes 2L and X is strongly supported by all analyses, and the small region on 2R is suggestive: these three regions are our candidate “speciation islands.” Using the HMM, we estimated the sizes of these regions to be 2,160 kb, 566 kb, and 37 kb for Chromosomes 2L, X, and 2R, respectively. We expect these values to be underestimates for the regions on 2L and X because some heterochromatic portions of the neighboring centromeres have not been assembled. The number of predicted genes in each chromosomal region is 50 in 2L, 12 in X, and five in 2R.

We directly assayed sequence variation from the islands in a larger sample to verify the contrast between these regions and the rest of the genome. Divergent regions were compared both to sequences from nearby loci that did not fall within the island of differentiation and to additional control sequences on Chromosome 3R. Sequenced loci are indicated in Figure 1, and sample sizes and sequence statistics are listed for all loci in Table 1. Fully supporting inferences from the whole-genome analysis, sequences from differentiated regions on Chromosomes 2L and X contained fixed differences and no shared polymorphisms, while adjacent control loci contained shared polymorphisms and no fixed differences (Table 1). This disparity between fixed and shared differences is highly significant at both regions (Chromosome 2L: seven fixed, none shared within the differentiated region, none fixed, ten shared outside the differentiated region, Fisher's exact test, *p* = 0.00005; Chromosome X: five fixed, none shared within the differentiated region, none fixed, four shared outside the differentiated region, Fisher's exact test, *p* = 0.008). The intron of the *P450–2* gene, in the divergent Chromosome X region, also had a 51-bp indel fixed between forms. The level of polymorphism within differentiated regions on Chromosomes 2L and X was low within each form (π ≤ 0.001 for all four loci), as would be expected if these regions had low rates of recombination because of proximity to centromeres. The nearby control region on X also had low polymorphism (Table 1), but showed no fixed differences.

**Table 1:**
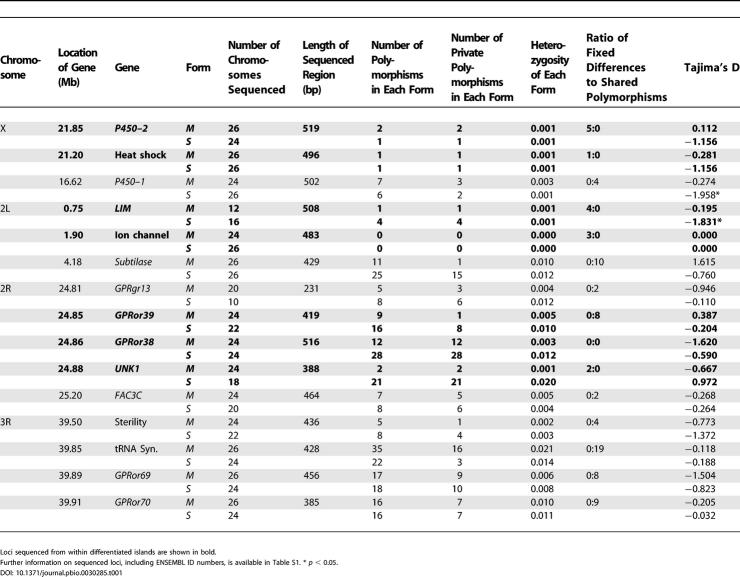
DNA Variation and Differentiation at Sequenced Loci

On Chromosome 2R we sequenced five loci: three loci inside the 37-kb region that was detected in both of our whole-genome analyses and two control loci adjacent to this region (one on either side; see Figure 1). Both control loci showed only shared polymorphisms and equal levels of nucleotide diversity between forms (Table 1). We found a single gene within the island that showed fixed differences and no shared polymorphisms between forms, similar to the loci on Chromosomes 2L and X. This gene (denoted *UNK1*) has no known function, and a BLASTn search yielded significant similarity only to an adjacent gene in *A. gambiae* that is also within the differentiated region but was not sequenced in our study. The two other genes within the island had no fixed differences, but all three loci had highly unequal levels of diversity between forms; *S* form mosquitoes showed up to 20 times the levels of nucleotide polymorphism as *M* form individuals. Gene *UNK1* showed the greatest asymmetry in polymorphism, with 21 SNPs in the *S* form sample and two in the *M* form sample. There is no evidence from tests of selection [31] that a recent sweep has occurred (Table 1), leaving the reasons for this asymmetry unclear.

Additional control sequences from Chromosome 3R showed shared polymorphisms between forms and no fixed differences, despite low levels of variability at some loci (Table 1). This highlights one possible complication of our analysis: reduced variability within forms may lead to fixed differences between forms in the absence of selection against hybrids [32]. Areas of low recombination that are exposed to repeated linked selection can show differentiation between populations, even in the face of high levels of gene flow [32,33]. Though there is little information on genomic recombination rates in *A. gambiae,* it is likely, as in *Drosophila,* that areas adjacent to centromeres have reduced recombination; these regions have high numbers of DNA repeats and low gene density [34]. The lack of fixed differences in our control regions adjacent to centromeres and the fixed differences we found in the middle of Chromosome 2R both refute the idea that recombination alone is responsible for the observed pattern of differentiation. We further tested for the effect of reduced recombination in causing the observed pattern through coalescent simulations. We used the program MS [35] to generate coalescent genealogies under a Wright–Fisher symmetric island model of gene flow between two subpopulations (cf. [33]). Estimates of the migration rate between forms in Cameroon were taken from the study by Wondji et al. [26] (we used the most conservative estimate, 4*N_e_m* = 10). We simulated two types of loci: one with levels of nucleotide polymorphism and recombination typical of most of our control regions, and one with 10-fold reductions in effective population size and recombination rate typical of most of our differentiated regions (see Materials and Methods). After generating 10,000 coalescent genealogies we did not observe a single instance of fixed differences in the absence of shared polymorphisms between subpopulations for our simulated control loci. Conversely, for our simulated differentiated loci we observed 32 instances where this occurred (*p* = 0.0032). Conservatively considering the two sequenced genes within each of our Chromosome 2L and Chromosome X islands as single loci, the probability of sequencing loci from both regions and observing fixed differences without shared polymorphisms—even with a much reduced effective population size—is *p* < 0.0001. It is therefore unlikely that decreased variability alone is responsible for the observed differences between forms.

## Discussion

Our genome-wide array analysis and our analysis of sequence polymorphism clearly show that differentiation between the *M* and *S* forms of *A. gambiae* is only present in a few regions of the genome. Although some of this similarity could be due to ancestral polymorphism, several lines of evidence support the hypothesis of substantial current gene flow between *A. gambiae M* form and *A. gambiae S* form*.* Previous studies have documented between-form mating of *A. gambiae* in nature (1.2% of scorable matings) [20,21], and hybrid *M/S* genotypes have been found (1.1% of larvae and 0.3% of adults in a population where *M* and *S* are sympatric) [36]. A previous survey using microsatellite loci of *M* and *S* forms in Cameroon found *F_st_* values between forms that were consistent with substantial migration rates (calculating 4*N_e_m* from the average *F_st_* reported in [26] yields 10 < 4*N_e_m* < 47). Microsatellite *F_st_* values are currently being calculated for the mosquitoes used in our study, with comparable results (F. Tripet, unpublished data).

In contrast to the low levels of differentiation found throughout most of the genome, our array experiments revealed three small regions to be significantly differentiated between forms. Sequences from islands on Chromosomes 2L and X contain 13 fixed differences and no shared polymorphisms. One gene within the third island, a 37-kb region on Chromosome 2R, also shows only fixed differences between forms.

In the early stages of divergence, above-average differentiation is expected between regions of low recombination. We conducted coalescent simulations to test whether the observed differentiation on Chromosomes 2L and X could plausibly result from neutral scenarios. Rejection of this neutral hypothesis (*p* < 0.0001) suggests that differentiation in these regions is due to selection against hybrid genotypes during backcrossing. This conclusion supports the prediction that when gene flow is present, differentiation between incipient species can be limited to small regions surrounding isolating genes [37]. No intrinsic postzygotic isolation has been found between forms, so we expect that the genes in these speciation islands are responsible for the observed prezygotic isolation or cause postzygotic isolation in nature (e.g., through ecological maladaptation, sexual isolation of hybrids from both parent species, or other causes).

Recombination between genes responsible for assortative mating and postzygotic isolation is thought to be a major barrier to speciation in the presence of gene flow [38]. Recent work has shown that inversions may facilitate speciation by creating linkage disequilibrium between these genes [39–41]. Although no direct information on recombination rates in these islands is available, the low polymorphism, high repeat density [34], and low gene density [34] within them suggest that regions near the centromeres have reduced recombination. This reduced recombination may create linkage disequilibrium between isolating factors, although the speciation islands we detected are so small that there are few genes within them to link together. Further investigation is necessary to determine whether our speciation islands contain co-adapted gene complexes, or whether they contain single loci experiencing divergent selection.

In the *A. gambiae* sensu lato species complex, overlapping distributions of partially isolated taxa are the rule and not the exception. *A. gambiae M* form and *A. gambiae S* form are broadly sympatric, and they are also sympatric with their two closest sibling species, *A. merus* and *A. arabiensis* [42], which are only partially isolated from the *A. gambiae* forms [16]. Mapping genomic differentiation between other members of this species complex will inform the study of speciation and adaptive radiation by showing whether there are consistent patterns of genomic differentiation between these species, how these speciation islands may change in size or number with time, and what genes within them are responsible for reproductive isolation.

## Materials and Methods

### DNA labeling and microarray hybridization

Mosquitoes were collected in the towns of Buea (one *M* form, one *S* form), Mutanguene (one *M* form, two *S* forms), and Tiko (five *M* forms, four *S* forms), in Cameroon in 2003 by the lab of G. C. Lanzaro (Department of Entomology, University of California, Davis, California, United States), who graciously shared samples for this study. DNA was extracted following Post et al. [43], and standard PCR diagnostics were used to differentiate *A. gambiae* from *A. arabiensis* [44] and to differentiate *A. gambiae M* and *S* forms [18]. Polytene chromosome preparation and analysis were as described in Hunt et al. [45]. Extracted DNA was labeled with an Invitrogen Bioprime Labling Kit (Invitrogen, Carlsbad, California, United States), following Borevitz et al. [29]. Isolated DNA in water (1,200 ng) was added on ice to 120 μl of random primers to a total volume of 200 μl. This solution was denatured in a 95 °C water bath, cooled on ice, then added to Klenow polymerase (6 μl), and dNTPs (30 μl). Incubation at 25 °C overnight resulted in small biotinylated oligos of approximately 50 bp. DNA was precipitated by adding 20 μl of 3 M NaOH and 400 μl of cold 100% EtOH. This solution was frozen at −80 °C for 15 min and centrifuged at 15,000*g* for 10 min, and the pelletized DNA was dried and resuspended in 50 μl of ddI H_2_O. Microarrays were hybridized by the University of California at Davis School of Medicine Microarray Core Facility using genomic DNA in place of cDNA in the standard Affymetrix protocol (Affymetrix, Santa Clara, California, United States).

### Microarray analysis

Raw hybridization intensities were normalized in R via RMA [46] using the Bioconductor Affy package [47] (http://www.bioconductor.org). Each probe on the array was blasted against the most recent *A. gambiae* genome assembly in order to remove probes with more than one exact match and to remap all probes onto the current assembly. We considered probes with two tailed *t*-test *p-*values less than 0.01 to contain SNPs between forms (1,577 total probes; this expectation was based on previous studies that validated the method [28,29], and was verified for our samples by sequence data discussed below). To test for regional differentiation, the chromosomal positions of probes with *p* < 0.01 were considered. To detect regional clustering of these probes, a sliding window of 300 probes was moved 30 probes at each step, and a χ^2^ test was performed to test whether the number of significant probes in each window was more than that expected by chance. Four regions were significant in this analysis at *p* < 0.05 after Bonferroni correction for the number of windows tested per chromosome. Three of these regions were robust to varying window sizes (data not shown). These analyses were carried out on each chromosome individually; the average window size was approximately 500 kb, with a range of approximately 60–2,000 kb, depending on the density of probes in each region. Significant probes could be more clustered than random because of the shared history of linked probes, so the sliding window analysis was repeated on 1,000 permuted datasets to test the effect of short-range linkage disequilibrium on our conclusions. For each permutation, probesets were reshuffled, but probes within a probeset remained associated. The 300-probe window with the highest number of significant probes in each permutation was recorded, and significance was assigned based on the number of permutations containing a window with differentiation equal to the observed value. As an additional test, we constructed a HMM [48] to segment each chromosome into introgressed and differentiated regions. Transition and emission probabilities of the HMM were estimated by expectation maximization; hidden states were then inferred using the Viterbi algorithm in Matlab (The MathWorks, Natick, Massachusetts, United States). The three divergent regions together are estimated to be approximately 2,740 kb, which is 1.2% of the assembled genome. All significant overlapping probes were combined in both analyses to control for nonindependence (two overlapping probes with *p* < 0.01 counted as one observation). Nonindependence could also arise because of deletions covering whole probesets; deletions were characterized by low *p*-values throughout a probeset, and were collapsed into one observation for the analysis. The data shown in Figure 1 were corrected for both overlapping probes and deletions in candidate regions. Excel files of normalized data for each chromosome are available from the authors upon request.

### Sequencing

All products were sequenced in both directions; see Table 1 for sequence lengths and sample sizes for each sequence. All individuals used in the whole-genome analysis were included, and sample sizes were increased to include additional samples collected in the same locations at the same time. Sequence traces were assembled and edited in CodonCode (http://www.codoncode.com, which uses ABI quality scores and Phred/Phrap to call bases, find heterozygous SNPs, and correct for heterozygous indels. Analysis of polymorphism and divergence was done in DNAsp (http://www.ub.es/dnasp/). The regions we sequenced covered nine probes with uncorrected *p-*values less than 0.01. The expected nucleotide difference was found in seven probes (78%), and one of the remaining two probes overlapped with a probe that contained the expected difference, highlighting the nonindependence of overlapping probes (which we corrected for in our whole-genome analysis). Because our samples were potentially heterozygous, we expected probes with low *p*-values to be either fixed differences or nucleotides with highly differentiated frequencies between forms. Sequencing of these six probes verified this expectation, as the detected nucleotide difference was either fixed between forms, or nearly fixed with either one or two heterozygous individuals for the rare allele.

### Coalescent simulations

Using the program MS [35], we generated coalescent genealogies with samples drawn from two subpopulations exchanging migrants. As with the structure of most of our sequenced loci, we simulated 26 alleles sampled from one population and 24 alleles sampled from the other. We conditioned the simulation of our control loci on the number of segregating sites observed at the Chromosome 2L control locus (*S* = 27) and the differentiated loci on the 2L loci within the island (*S* = 12). We estimated migration to be 4*N_e_m* = 10 for the control loci and 4*N_e_m* = 1 for the differentiated loci [26]. Because recombination rates are not known, we used the typical value of *r* = 1 × 10^−8^ for recombination per site in control regions (4*N_e_r* = 2; *r* = 1 × 10^–8^; *N_e_* = 100,000; 500 bases per locus) and *r* = 1 × 10^–9^ for recombination per site in differentiated regions (4*N_e_r* = 0.04; *r* = 1 × 10^−9^; *N_e_* = 10,000; 1,000 bases per locus). All simulations were run 10,000 times, where the output of MS was parsed to count the number of fixed and shared polymorphisms for each run. To obtain the *p-*value associated with observing two loci with fixed differences and no shared polymorphisms, we sampled two loci for each of the 10,000 iterations and counted the number of times both showed this pattern.

## Supporting Information

Table S1 Additional Information on Sequenced Loci (23 KB XLS).

### Accession Numbers

The GenBank (http://www.ncbi.nlm.nih.gov/Genbank) accession numbers for the gene sequences discussed in this paper areAY825543–AY825922 and DQ080663–DQ080909.

## Abbreviations

2L: left arm of Chromosome 2
2R: right arm of Chromosome 2
*A. gambiae*: *Anopheles Gambiae* sensu strictu
bp: base pair(s)
HMM: hidden Markov model
kb: kilobase(s)
SFP: single-feature polymorphism
SNP: single nucleotide polymorphism
